# Supplementary material for: Pathophysiological Significance of Neutrophilic Transfer RNA-Derived Small RNAs in Asymptomatic Moyamoya Disease
Source: Cells. 2021 May 1;10(5):1086. doi: 10.3390/cells10051086 (PMC8147334; doi:10.3390/cells10051086)

2D Structure	Local AU	Position

```
133                              8mer-1a   168
 5'-gaATGTGGTTACTTCCTACTGTAGGGTAGCGGAAAa-3' utr
      |:|||:| |    |:|||::|   : :||||||
 3'-uuUGCACUAUU----GGUGAUGU---GAUGCCUUUg-5' tRF
                     16151413      7 6 5 4 3 2
                   3' Pairing         Seed
```

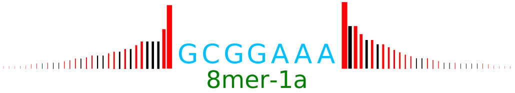

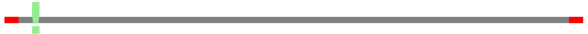

Supplement: Supplementary file 1 [file cells-10-01086-s001.zip › cells-1149161 3rd supplementary materials/Data Supplements/Data Supplement V.zip!/Data supplement 6-mRNAs targets of tsRNAs/tsRNA-Val-AAC-017/Diagrams/tRF-Val-AAC-017_vs_ENST00000256442(CCNB1).pdf]
